# Supplementary material for: Heat adaptation of phage T7 under an extended genetic code
Source: Virus Evol. 2021 Dec 1;7(2):veab100. doi: 10.1093/ve/veab100 (PMC8923235; doi:10.1093/ve/veab100)
Supplement: veab100_Supp [file veab100_supp.zip › Sequencing_Analysis.zip!/Sequencing_Analysis/S3/evidence/RA_21.html]

BRESEQ :: Evidence


| Read alignment evidence... | | | | | | | | | | | |
| --- | --- | --- | --- | --- | --- | --- | --- | --- | --- | --- | --- |
|  | seq id | position | | ref | new | freq | score (cons/poly) | reads | annotation | genes | product |
| \* | NC\_001604.1 | 19,319 | 0 | T | C | 23.2% | 2436.2 / inf | 1547 | intergenic (–/–) | *–/–* | –/– |
| Reads supporting (aligned to +/- strand):  **ref** base T (598/579);  **new** base C (192/164);  **total** (790/757) | | | | | | | | | | | |
| Fisher's exact test for biased strand distribution *p*-value = 3.04e-01 | | | | | | | | | | | |
| Kolmogorov-Smirnov test that lower quality scores support variant *p*-value = 1.00e+00 | | | | | | | | | | | |

Only 100 of 1557 total aligned reads displayed.  

`AGGCCTCAAGGTCGAACGTGATGATTCCGTAGCGAAGTCTAAAGCCAGCGGCAATGGCTCCGCTCGTATGAAATCTTCCATCCGTAAGTCCGCATTTGGAGGTAAGAAGTGATGTCTGAGTTCACATGTGTGGAGGCTAAGAGTCGCTTCCGTGCAATCCGGTGGACTGTGGAACACCTTGGGTTGCCTAAAGGATTCGAAGGACACTTTGTGGGCTACAGCCTCTACGTAGACGAAGTGATGGACATGTCTGGTTGCCGTGAAGAGTACATTCTGGACTCTACCGGAAAACATGTAGCGTACTTCGCGTGGTGCGTAAGCTGTGACATTCACCACAAAGGAGACATTCTGGATGTAACGTCCGTTGTCATTAATCCTGAGGCAGACTCTAAGGGCTTACAGCGATTCCTAGCGAAACGCTTTAAGTACCTTGCGGAACTCCACGATTGCGATTGGGTGTCTCGTTGTAAGCATGAAGGCGAGACAATGCGTGTATACTTTAAGGAGGTATAAGTTATGGGTAAGAAAGTTAAGAAGGCCGTGAAGAAAGTCACCAAGTCCGTTAAGAAAGTCGTTAAGGAAGGGGCTCGTCCGGTTAAAC  >  NC_001604.1/19019‑19619`  

                                                                                                                                                                                                                                                                                                            `|`                                                                                                                                                                                                                                                                                                              
   `ccTCAAGGTCGAACGTGATGATTCCGTAGCGAAGTCTAAAGCCAGCGGCAATGGCTCCGCTCGTATGAAATCTTCCATCCGTAAGTCCGCATTTGGAGGTAAGAAGTGATGTCTGAGTTCACATGTGTGGAGGCTAAGAGTCGCTTCCGTGCAATCCGGTGGACTGTGGAACACCTTGGGTTGCCTAAAGGATTCGAAGGACACTTTGTGGGCTACAGCCTCTACGTAGACGAAGTGATGGACATGTCTGGTTGCCGTGAAGAGTACATTCTGGACTCTACCGGAAAACATGTAGCGTACt                                                                                                                                                                                                                                                                                                           >  1:262517/1‑301 (MQ=255)`  
    `cTCAAGGTCGAACGTGATGATTCCGTAGCGAAGTCTAAAGCCAGCGGCAATGGCTCCGCTCGTATGAAATCTTCCATCCGTAAGTCCGCATTTGGAGGTAAGAAGTGATGTCTGAGTTCACATGTGTGGAGGCTAAGAGTCGCTTCCGTGCAATCCGGTGGACTGTGGAACACCTTGGGTTGCCTAAAGGATTCGAAGGACACTTTGTGGGCTACAGCCTCTACGTAGACGAAGTGATGGACATGTCTGGTTGCCGTGAAGAGTACATTCTGGACTCTACCGGAAAACATGTAGCGTACtt                                                                                                                                                                                                                                                                                                          <  1:2870/301‑1 (MQ=255)`  
    `cTCAAGGTCGAACGTGATGATTCCGTAGCGAAGTCTAAAGCCAGCGGCAATGGCTCCGCTCGTATGAAATCTTCCATCCGTAAGTCCGCATTTGGAGGTAAGAAGTGATGTCTGAGTTCACATGTGTGGAGGCTAAGAGTCGCTTCCGTGCAATCCGGTGGACTGTGGAACACCTTGGGTTGCCTAAAGGATTCGAAGGACACTTTGTGGGCTACAGCCTCTACGTAGACGAAGTGATGGACATGTCTGGTTGCCGTGAAGAGTACATTCTGGACTCTACCGGAAAACATGTAGCGCACtt                                                                                                                                                                                                                                                                                                          >  1:235008/1‑301 (MQ=255)`  
              `aaCGTGATGATTCCGTAGCGAAGTCTAAAGCCAGCGGCAATGGCTCCGCTCGTATGAAATCTTCCATCCGTAAGACCGCATTTGGAGGTAAGAAGTGATGTCTGAGTTCACATGTGTGGAGGCTAAGAGTCGCTTCCGTGCAATCCGGTGGACTGTGGAACACCTTGGGTTGCCTAAAGGATTCGAAGGACACTTTGTGGGCTACAGCCTCTACGTAGACGAAGTGATGGACATGTCTGGTTGCCGTGAAGAGTACATTCTGGACTCTACCGGAAAACAAGTAGCGTACTCCGCggggtgg                                                                                                                                                                                                                                                                                                >  1:340352/1‑300 (MQ=255)`  
                 `ggacagatTCCGTAGCGAAGTCGAAAGTCAGCGGCAATGGCTCCGCTCGTATGAAATCTTCAATACGTAAGTCCGCATTTGGAGGTAAGAAGTGATGTCTGAGTTCACATGTGTGGAGGCTAAGAGTCGCTTCCGTGCAATCCGGTGGACTGTGGAACACCTTGGGTTGCCTAAAGGATTCGAAGGACACTTTGTGGGCTACAGCCTCTACGTAGACGAAGTGATGGACATGTCTGGTTGCCGTGAAGAGTACATTCTGGACTCTACCGGAAAACATGTAGCGTACTTCGCGTGGTGCGTa                                                                                                                                                                                                                                                                                             <  1:217963/296‑1 (MQ=255)`  
                   `gatgatTCCGTAGCGAAGTCTAAAGCCAGCGGCAATGGCTCCGCTCGTATGAAATCGTCCATCCGTAAGTCCGCATTTGGAGGTAAGAAGTGATGTCTGAGTTCACATGTGTGGAGGCTAAGAGTCGCTTCAGTGCAATCCGGTGGACTGTGGAACACCTTGGGTTGCCTAAAGGATTCGAAGGACACTTTGTGGGCTACAGCCTCTACGTAGACGAAGTGATGGACATGTCTGGTTGCCGTGAAGAGTACATTCTGGACTCTACCGGAAAACATGTAGCGTACTTCGCGTGGTGCGTAAg                                                                                                                                                                                                                                                                                           <  1:162182/301‑1 (MQ=255)`  
                    `atgatTCCGTAGCGAAGTCTAAAGCAAGCGGCAATCGCTCCGATCGTATGAAATCTTCCATCCGTAAGTCCGGATTTGGAGGTAAGGAGTGATGTCTGAGTTCACATGTGTGTAGGCTAAGAGTCGCTTCCGTGCAATCCGGTGGACTGTGGAAAACCTTGGGTTGCCTAAAGGATTCGAAGGACACTTTGTGGGCTACAGCCGCTACGTAGACGAAGTGATGGACATGTCTGGTTGCCGTGAAGAGTACATTCTGGACTCTACCGGAAAACATGTAGCGCACTTCGCGTGGTGCGTAAGc                                                                                                                                                                                                                                                                                          <  1:284891/301‑1 (MQ=255)`  
                      `gatTCCGTAGCGAAGTCTAAAGCCAGCGGCAATGGCTCCGCTTGTATGAAATCTTCCATCCGTAAGTCCGCATTAGGAGGTAAGAAGTGATGTCTGAGTTCACATGTGTGGAGGCTAAGAGTCGCTTCCGTGCAATCCGGTGGACTGTGGAACACCTTGGGTTGCCTAAAGGATTCGAAGGACACTTTGTGGGCTACAGCCTCTACGTAGACGAAGTGCTGGACAAGTCTGGGTGCCGTGCAGAGTACATTCTGGAGTCTACCGGGAACCAAGTAGCGCACTTCGAgtggtgactgtctct                                                                                                                                                                                                                                                                                        >  1:92594/1‑292 (MQ=255)`  
                              `aGCGAAGTCTAAAGCCAGCGGCAATGGCTCCGCTCGTATGAAATCTTCCATCCGTAAGTCCGCATTTGGAGGTAAGAAGTGATGTCTGAGTTCACATGTGTGGAGGCTAAGAGTCGATTCCGTGCAATCCGGTGGACTGTGGAACACCTTGGGTTGCCTAAAGGATTCGAAGGACACTTTGTGGGCTACAGCCTCTACGTAGACGAAGTGATGGACATGTCTGGTTGCCGTGAAGAGTACATTCTGGACTCTACCGGAAAACATGTAGCGTACTTCGCGTGGTGCGTAAGCTGTGACATTc                                                                                                                                                                                                                                                                                <  1:124201/301‑1 (MQ=255)`  
                                    `gTCTAAAGCCAGCGGCAATGGCTCCGCTCGTATGAAATCTTCCATCCGTAAGTCCGCATTTGGAGGTAAGAAGTGATGTCTGAGTTCACATGTGTGGAGGCTAAGAGTCGCTTCCGTTCAATCCGGTGGACTGTGGAACACCTTGGGTTGCCTAAAGGATTCGAAGGACACTTTGTGGGCTACAGCCTCTACGTAGACGAAGTTATGGACATGTCTGGTTGCCGTGAAGAGTACATTCTTGACTCTACCGGAAAACATGTAGCGCACTTCGCGTGGTGCGTAAGCTGTGACATTCCCcaca                                                                                                                                                                                                                                                                          >  1:65751/1‑301 (MQ=255)`  
                                    `gTCTAAAGCCAGCGGCAATGGCTCCGCTCGTATGAAATCTTCCATCCGTAAGTCCGCATTTGGAGGTAAGAAGTGATGTCTGAGTTCACATGTGTGGAGGCTAAGAGTCGCTTCCGTGCAATCCGGTGGACTGTGGAACACCTTGGGTTGCCTAAAGGATTCGAAGGACACTTTGTGGGCTACTGCCTCTACGTAGACGAAGTGATGGACATGTCTGGTTGCCGTGAAGAGTACATTCTGGACTCTACCGGAAAACATGTAGCGCACTTCGCGTGGTGCGTAAGCTGTGACATTCACcaca                                                                                                                                                                                                                                                                          >  1:312807/1‑301 (MQ=255)`  
                                    `gTCTAAAGCCAGCGGCAATGGCTCCGCTCGTATGAAATCTTCCATCCGTAAGTCCGCATTTGGAGGTAAGAAGTGATGTCTGAGTTCACATGTGTGGAGGCTAAGAGTCGCTTCCGTGCAATCCGGTGGACTGTGGAACACCTTGGGTTGCCTAAAGGATTCGAAGGACACTTTGTGGGCTACAGCCTCTACGTAGACGAAGTGATGGACATGTCTGGTTGCCTTGAAGAGTACATTCTGGACTCTACCGGAAAACATGTAGCGTACTCCGCGTGGTTCGTAAGCTGTGACATTCACcaca                                                                                                                                                                                                                                                                          >  1:185998/1‑301 (MQ=255)`  
                                    `gTCTAAAGCCAGCGGCAATGGCTCCGCTCGTATGAAATCTTCCATCCGTAAGTCCGCATTTGGAGGTAAGAAGTGATGTCTGAGTTCACATGTGTGGAGGCTAAGAGTCGCTTCCGTGCAATCCGGTGGACTGTGGAACACCTTGGGTTGCCTAAAGGATTCGAAGGACACTTTGTGGGCTACAGCCTCTACGTAGACGAAGTGATGGACATGTCTGGTTGCCGTGAAGAGTACATTCTGGACTCTACCGGAAAACATGTAGCGTACTTCGCGTGGTGCGTAAGCTGTGACATTCACcaca                                                                                                                                                                                                                                                                          >  1:169822/1‑301 (MQ=255)`  
                                    `gTCTAAAGCCAGCGGCAATGGCTCCGCTCGTATGAAATCTTCCATCCGTAAGTCCGCATTTGGAGGTAAGAAGTGATGTCTGAGTTCACATGTGTGGAGGCTAAGAGTCGCTTCCGTGCAATCCGGTGGACTGTGGAACACCTTGGGTTGCCTAAAGGATTCGAAGGACACTTTGTGGGCTACAGCCTCTACGTAGACGAAGTGATGGACATGTCTGGTTGCCGTGAAGAGTACATTCTGGACTATACCGGACAACATGTAGCGTACTTCGCGTGGTGCGTAAGCTGTGACATTcaccctg                                                                                                                                                                                                                                                                          >  1:299247/1‑298 (MQ=255)`  
                                        `aaaGGCAGCGGCAATGGCTCCGCTCGTATGAAATCTTCCATCCGTAAGTCCGCATTTGGAGGTAAGAAGTGATGTCTGAGTTCACATGTGTGGAGGCTAAGAGTCGCTTCCGTGCAATCCGGTGGACTGTGGAACACCTTGGGTTGCCTAAAGGATTCGAAGGACACTTTGTGGGCTACAGCCTCTACGTAGACGAAGTGATGGACATGTCTGGTTGCCGTGAAGAGTACATTCTGGACTCTACCGGAAAACATGTAGCGTACTTCGCGTGGTGCGTAAGCTGTGACATTCACCACAAAgg                                                                                                                                                                                                                                                                      <  1:318982/301‑1 (MQ=255)`  
                                            `ccAGCGGCAATGGCTCCGCTCGTATGAAATCTTCCATCCGTAAGTCCGCATTTGGAGGTAAGAAGTGATGTCTGAGTTCACATGTGTGGAGGCTAAGAGTCGCTTCCGTGCAATCCGGTGGACTGTGGAACACCTTGGGTTGCCTAAAGGATTCGAAGGACACTTTGTGGGCTACAGCCTCTACGTAGACGAAGTGATGGACATGTCTGGTTGCCGAGAAGAGTACATTCTGGACTCTACCGGAAAACATGTAGCGTACTTCGCGTGGTGCGTAAGCTGTGACATTCACCACAAAGGAGAc                                                                                                                                                                                                                                                                  <  1:292613/301‑1 (MQ=255)`  
                                                `cGGCAATGGCTCCGCTCGTATGAAATCTTCCATTCGTAAGTCCGCATTTGGAGGGAATAAGTGATGTCTGAGTTAACATGTGTGGAGGCTAAGAGTCGCTTCCGTGCAATCAGGTGGACTGTGGAACACCTTGGGTTGCCTAAAGGATTCGAAGGATACTTTGTGGGCTACAGCCTCTACGTAGACGAAGTGATGGACATGTCTGGTTGCCGTGAAGAGTACATTCTGGACTCTACCGGAAAACATGTAGCGTACTTCGCGTGGTGCGTAAGCTGTGACATTCACCACAAAGGAGACATTc                                                                                                                                                                                                                                                              <  1:50517/301‑1 (MQ=255)`  
                                                     `agggccaggcgtgTATGAAATCGTCCATCCGTAAGGCCGCATTTGGAGGTAAGAAGTGATGTCTGAGTTCACATGTGTGGAGGCTAAGAGTCGCTTCCGTGCAATCCGGTGGACTGTGGAACTCCTTGGGTTGCCTAAAGGATTCGAAGGACACTTTGTGGGCTACAGCCTCTACGTAGACGAAGTGATGGACATGTCTGGTTGCCGTGAAGAGTACATTCTGGACTCTACCGGAAAACATGTAGCGTACTTCGCGTGGTGCGTAAGCTGTGACATTCACCACAAAGGAGACATTCTGGAt                                                                                                                                                                                                                                                         <  1:176749/289‑1 (MQ=255)`  
                                                       `ggCTCCGCTCGTATGAAATCTTCCATCCGTAAGTCCGCATTTGGAGGTAAGAAGTGATGTCTGAGTTCACATGTGTGGAGGCTAAGAGTCGCTTCCGTGCAATCCGGTGGACTGTGGAACACCTTGGGTTGCCTAAAGGATTCGAAGGACACTTTGTGGGCTACAGCCTCTACGTAGACGAAGTGATGGACATGTCTGGTTGCCGTGAAGAGTACATTCTGGACTCTACCGGAAAACATGTAGCGCACTTCGCGTGGTGCGTAAGCTGTGACATTCACCACAAAGGAGACATTCTGGATGt                                                                                                                                                                                                                                                       >  1:5705/1‑301 (MQ=255)`  
                                                           `ccGCTCGTATGAAATCTTCCATCCGTAAGGCCGCATTTGGAGGTAAGAAGTGATGTCTGAGTTCACATGTGTGGAGGCTAAGAGTCGCTTCCGTGCAATCCGGTGGACTGTGGAACACCTTGGGTTGCCTAAAGGATTCGAAGGACACTTTGTGGGCTACAGCCTCTACGTAGACGAAGTGATGGACATGTCTGGTTGCCGTGAAGAGTACATTCTGGACTCTACCGGAAAACATGTAGCGTACTTCGCGTGGTGCGTAAGCTGTGACATTCACCACAAAGGAGACATTCTGGATGTAACg                                                                                                                                                                                                                                                   <  1:179923/301‑1 (MQ=255)`  
                                                                 `gTATGAAATCTTCCATCCGTAAGTCCGCATTTGGAGGTAAGAAGTGATGTCTGAGTTCACATGTGTGGAGGCTAAGAGTCGCTTCCGTGCAATCCGGTGGACTGTGGAACACCTTGGGTTGCCTAAAGGATTCGAAGGACACTTTGTGGGCTACAGCCTCTACGTAGACGAAGTGATGGACATGTCTGGTTGCCGTGAAGAGTACATTCTGGACTCTACCGGAAAACATGTAGCGCACTTCGCGTGGTGCGTAAGCTGTGACATTCACCACAAAGGAGACATTCTGGATGTAACGTCCGtt                                                                                                                                                                                                                                             >  1:275055/1‑301 (MQ=255)`  
                                                                  `tATGAAATCTTCCATCCGTAAGTCCGCATTTGGAGGTAAGAAGTGATGTCTGAGTTCACATGTGTGGAGGCTAAGAGTCGCTTCCGTGCAATCCGGTGGACCGTGGAACACCTTGGGTTGCCTAAAGGATTCGAAGGACACTTTGTGGGCTACAGCCTCTACGTAGACGAAGTGATGGACATGTCTGGTTGCCGTGAAGAGTACATTCTGGACTCTACCGGAAAACATGTAGCGTACTTCGCGTGGTGCGTAAGCTGTGACATTCACCACAAAGGAGACATTCTGGATGTAACATCCGCTg                                                                                                                                                                                                                                            >  1:108799/1‑301 (MQ=255)`  
                                                                          `cTTCCATCCGTAAGTCCGCATTTGGAGGTAAGGAGTGATGTCTGAGTTCAAATGTGTGGAGGCTAAGAGTCGCTTCCTTGCAATCCGGTGGACTGTGGAACACCTTGGGTTGCCTAAAGGATTCGAAGGACACTTTGTGGGCTACAGCCTCTACGTAGACGAAGTGATGGACATGTCTGGTTGCCGTGAAGAGTACATTCTGGACTCTACCGGAAAACATGTAGCGTACTTCGCGTGGTGCGTAAGCTGTGACATTCACCACAAAGGAGACATTCTGGATGTAACGTCCGTTGTCATTAAt                                                                                                                                                                                                                                    <  1:346724/301‑1 (MQ=255)`  
                                                                                        `tCCGCATTTGGAGGTAAGAAGTGATGTCTGAGGTCACATGTGTGGAGGCTAAGAGTCGCTTCCGTGCAATCCGGTGGACTGTGGAACACCTTGGGTTGCCTAAAGGATTCGAAGGACACTTTGTGGGCTACAGCCTCTACGTAGACGAAGTGATGGACATGTCTGGTTGCCGTGAAGAGTACATTCTGGACTCTACCGGAAAACATGTAGCGTACTTCGCGTGGTGCGTAAGCTGTGACATTCACCACAAAGGAGACATTCTGGATGTAACGTCCGTTGTCATTAATCCTGAGGCAGActc                                                                                                                                                                                                                      <  1:282211/301‑1 (MQ=255)`  
                                                                                         `ccGCATTTGGAGGTAAGAAGTGATGTCTGAGTTCACATGTGTGGAGGCTAAGAGTCGCTTCCGTGCAATCCGGTGGACTGTGGAACACCTTGGGTTGCCTAAAGGATTCGAAGGACACTTTGTGGGCTACAGCCTCTACGTAGACGAAGTGATGGACATGTCTGGTTGCCGTGAAGAGTACATTCTGGACTCTACCGGAAAACATGTAGCGCACTTCGCGTGGTGCGTAAGCTGTGACATTCACCACAAAGGAGACATTCTGGATGTAACGTCCGTTgctgtctcttatacacatctccga                                                                                                                                                                                                                     >  1:76985/1‑278 (MQ=255)`  
                                                                                             `aTTTGGAGGTAAGAAGTGATGTCTGAGTTCACATGTGTGGAGGCTAAGAGTCGCTTCCGTGCAATCCGGTGGACTGTGGAACACCTTGGGTTGCCTAAAGGATTCGAAGGACACTTTGTGGGCTACAGCCTCTACGTAGACGAAGTGATGGACATGTCTGGTTGCCGTGAAGAGTACATTCTGGACTCTACCGGAAAACATGTAGCGTACTTCGCGTGGTGCGTAATCTGTGACATTCACCACAAAGGAGACATTCTGGATGTAACGTCCGTTGTCATTAATCCTGAGGCAGACTCTAAgg                                                                                                                                                                                                                 >  1:205179/1‑301 (MQ=255)`  
                                                                                                     `gaacagagggATGTCTGGGTTCACATGTGTGGAGGCTAAGAGTCGATTCCGTGCAATCCGGTGGACTGTGGAACACCTTGGGTTGCCTAAAGGATTCGAAGGACACTTTGTGGGCTACAGCCTCTACGTAGACGAAGTGATGGACATGTCTGGTTGCCGTGAAGAGTACATTCTGGACTCTACCGGAAAACATGTAGCGTACTTCGCGTGGTGCGTAAGCTGTGACATTCACCACAAAGGAGACATTCTGGATGTAACGTCCGTTGTCATTAATCCTGAGGCAGACTCTAAGGGCTTACAg                                                                                                                                                                                                         <  1:36888/292‑1 (MQ=255)`  
                                                                                                           `agTGATGTCTGAGTTCACATGTGTGGAGGCTAAGAGTCGCTTCCGTGCAATCCGGTGGACTGTGGAACACCTTGGGTTGCCTAAAGGATTCGAAGGACACTTTGTGGGCTACAGCCTCTACGTAGACGAAGTGATGGACATGTCTGGTTGCCGTGAAGAGTACATTCTGGACTCTACCGGAAAACATGTAGCGTACTTCGCGTGGTGCGTAAGCTGTGACATTCACCACAAAGGAGACATTCTGGATGTAACGTCCGTTGTCATTAATCCTGAGGCAGACTCTAAGGGCTTACAGCGATTc                                                                                                                                                                                                   <  1:330254/301‑1 (MQ=255)`  
                                                                                                                     `gAGTTCACATGTGTGGAGGCTAAGAGTCGCTTCCGTGCAATCCGGTGGACTGTGGAACACCTTGGGTTGCCTAAAGGATTCGAAGGACACTTTGTGGGCTACAGCCTCTACGTAGACGAAGTGATGGACATGTCTGGTTGCCGTGAAGAGTACATTCTGGACTCTACCGGAAAACATGAAGCGTACTTCGCGTGGTGCGTAAGCTGTGACATTCACCACAAAGGAGACATTCTGGATGTAACGTCCGTTGTCATTAATCCTGAGGCAGACTCTCAggattaatgacaacggacgttacatc                                                                                                                                                                                         >  1:147805/1‑277 (MQ=255)`  
                                                                                                                       `gTTCACATGTGTGCAGGCTAAGAGTCGCTTCCGTGCAATCCGGTGGACTGTGGAACACCTTGGGTTGCCTAAAGGATTCGAAGGACACTTTGTGGGCTACAGCCTCTACGTAGACGAAGTGATGGACATGTCTGGTTGCCGTGAAGAGTACTTTCTGGACTCTACCGGAAAACATGTAGCGTACTTCGCGTGGTTCTTAAGCTGTGACATTCACCACAAAGGAGACATTCTGGATGTAACGTCCTTTGTCATTAATCCTGTGGCAGACTCTAAGCGCtgtctcttatacacctctccgagc                                                                                                                                                                                       >  1:131978/1‑278 (MQ=255)`  
                                                                                                                                `gagacaGGGTAAGAGTCGCTTCCGTGCAATCCGGTGGACTGTGGAACACCTTGGGTTGCCTAAAGGATTCGAAGGACACTTTGTTGGCTACAGCCTCTACGTAGACGAAGTGATGGACATGTCTGGTTGCCGTGAAGAGTACATTCTGGACTCTACCGGAAAACATGTAGCGTACTTCGCGTGGTGCGTAAGCTGTGACATTCACCACAAAGGAGACATTCTGGATGTAACGTCCGTTGTCATTAATCCTGAGGCAGACTCTAAGGGCTTACAGCGATTCCTAGCGAAACGCTTTAAGTAc                                                                                                                                                                              <  1:232184/296‑1 (MQ=255)`  
                                                                                                                                `agacagggCTAAGAGTCGCTTCCGTGCAATCCGGTGGACTGTGGAACACCTTGGGTTGCCTAAAGGATTCGAAGGACACTTTGTGGGCTACAGCCTCTACGTAGACGAAGTGATGGACATGTCTGGTTGCCGTGAAGAGTACATTCTGGACTCTACCGGAAAACATGTAGCGTACTTCGCGTGGTGCGTAAGCTGTGACATTCACCACAAAGGAGACATTCTGGATGTAACGTCCGTTGTCATTAATCCTGAGGCAGACTCTAAGGGCTTACAGCGATTCCTAGCGAAACGCTTTAAGTAc                                                                                                                                                                              <  1:88893/295‑1 (MQ=255)`  
                                                                                                                                 `gtgGAGGCTAAGAGTCGCTTCCGTGCAATCCGGTGGACTGTGGAACACCTTGGGTTGCCTAAAGGATTCGAAGGACACTTTGTGGGCTACAGCCTCTACGTAGACGAAGTGATGGACATGTCTGGTTGCCGTGAAGAGTACATTCTGGACTCTACCGGAAAACATGTAGCGTACTTCGCGTGGTGCGTAAGCTGTGACATTCACCACAAAGGAGACATTCTGGATGTAACGTCCGTTGTCATTAATCCTGAGGCAGACTCTAAGGGCTTACAGCGATTCCTAGCGAAACGCTTTAAGTAcc                                                                                                                                                                             <  1:238173/301‑1 (MQ=255)`  
                                                                                                                                  `tgGAGGCTAAGAGTCGCTTCCGTGCAATCCGGTGGACTGTGTAACACCTTGGGTTGCCTAAAGGATTCGAAGGTCACTTTGTGGGCTACAGCCTCTACGTAGACGAAGTGATGGACATGTCTGGTTGCCGTGAAGAGTACATTCTGGACTCTACCGGAAAACATGTAGCGTACTTCGCGTGGTGCGTAAGCTGTGACATTCACCACAAAGGAGACATTCTGGATGTAACGTCCGTTGTCATTAATCCTGAGGCAGACTCTAAGGGCTTACAGCGATTCCTAGCGAAACGCTTTAAGTACCt                                                                                                                                                                            <  1:315117/301‑1 (MQ=255)`  
                                                                                                                                    `gAGGCTAAGAGTCGCTTCCGTGCAATCCGGTGGACTGTGGAACACCTTGGGTTGCCTAAAGGATTCGAAGGACACTTTGTGGGCTACAGCCTCTACGTAGACGAAGTGATGGACATGTCTGGTTGCCGTGAAGAGTACATTCTGGACTCTACCGGAAAACATGTAGCGTACTTCGCGTGGTGCGTAAGCTGTGACATTCACCACAAAGGAGACATTCTGGATGTAACGTCCGTTGTCATTAATCCTGAGGCAGACTCTAAGGGCTTACAGCGATTCCTAGCGAAACGCTTTAAGTACCTTg                                                                                                                                                                          >  1:260166/1‑301 (MQ=255)`  
                                                                                                                                      `ggCTAAGAGTCGCTTCCGTGCAATCCGGTGGACTGTGGAACACCTTGGGTTGCCTAAAGGATTCGAAGGACACTTTGTGGGCTACAGCCTCTACGTAGACGAAGTGATGGACATGTCTGGTTGCCGTGAAGAGTACATTCTGGACTATACCGGAAAATATGTAGCGTACTTCGCGTGGTGCGTAAGCTGTGACATTCACCACAAAGGAGACATTCTGGATGTAACGTCCGTTGTCATTAATCCAGAGGCAGAATCTAAGGGCTTACAGCGATTACTAGCGAAACGCGTTAAGTACCTTGCg                                                                                                                                                                        >  1:129990/1‑301 (MQ=255)`  
                                                                                                                                                 `gtataagagacagccGGTGGACTGTGGAACACCTTGGGTTGCCTAAAGGATTCGAAGGACACTTTGTGGGCTACAGCCTCTACGTAGACGAAGTGATGGACATGTCTGGTTGCCGTGAATAGTACATTCTGGACTCTACCGGAAAACATGTAGCGTACTTCGCGTGGTGCGTAAGCTGTGACATTCACCACAAAGGAGACATTCTGGATGTAACGTCCGTTGTCATTAATCCTGAGGCAGACTCTAAGGGCTTACAGCGATTCCTAGCGAAACGCTTTAAGTACCTTGCGGAACTCCAcga                                                                                                                                                             <  1:32701/288‑1 (MQ=255)`  
                                                                                                                                                 `gCTTCCGTGCAATCCGGTGGACTGTGGAACACCTTGGGTTGCCTAAAGGATTCGAAGGACACTTTGTGGGCTACAGCCTCTACGTAGACGAAGTGATGGACATGTCTGGTTGCCGTGAAGAGTACATTCTGGACTCTACCGGAAAACATGTAGCGCACTTCGCGTGGTGCGTAAGCTGTGACATTCACCACAAAGGAGACATTCTGGATGTAACGTCCGTTGTCATTAATCCTGAGGCAGACTCTAAGGGCTTACAGCGATTCCTAGCGAAACGCTTTAAGTACCTTGCGGAACTCCAcga                                                                                                                                                             >  1:194912/1‑301 (MQ=255)`  
                                                                                                                                                  `cTTCCGTGCAATCCGGTGGACTGTGGAACACCTTGGGTTGCCTAAAGGATTCGAAGGACACTTTGTGGGCTACAGCCTCTACGTAGACGAAGTGATGGACATGTCTGGTTGCCGTGAAGAGTACATTCTGGACTCTACCGGAAAACATGTAGCGCACTTCGCGTGGTGCGTAAGCTGTGACATTCACCACAAAGGAGACATTCTGGATGTAACGTCCGTTGTCATTAATCCTGAGGCAGACTCTAAGGGCTTACAGCGATTCCTAGCGAAACGCTTTAAGTACCTTGCGGAACTCCAcgat                                                                                                                                                            >  1:69554/1‑301 (MQ=255)`  
                                                                                                                                                    `aaagagacagccGGTGGACTGTGGAACACCTTGGGTTGCCTAAAGGATTCGAAGGACACTTTGTGGGCTACAGCCTCTACGTAGACGAAGTGATGGACATGTCTGGTTGCCGTGAAGAGTACATTCTGGACTCTACCGGAAAACATGTAGCGTACTTCGCGTGGTGCGTAAGCTGTGACATTCACCACAAAGGAGACATTCTGGATGTAACGTCCGTTGTCATTAATCCTGAGGCAGACTCTAAGGGCTTACAGCGATTCCTAGCGAAACGCTTTAAGTACCTTGCGGAACTCCAcgattg                                                                                                                                                          <  1:253017/291‑1 (MQ=255)`  
                                                                                                                                                       `gTGCAATCCGGTGGACTGTGGAACACCTTGGGTTGCCTAAAGGATTCGAAGGACACTTTGTGGGCTACAGCCTCTACGTAGACGAAGTGATGGACATGTCTGGTTGCCGTGAAGAGTACATTCTGGACTCTACCGGAAAACATGTAGCGTACTTCGCGTGGTGCGTAAGCTGTGACATTCACCACAAAGGAGACATTCTGGATGTAACGTCCGTTGTCATTAATCCTGAGGCAGACTCTAAGGGCTTACAGCGATTCCTAGCGAAACGCTTTAAGTACCTTGCGGAACTCCAcgatctgtc                                                                                                                                                       >  1:335351/1‑296 (MQ=255)`  
                                                                                                                                                            `aTCCGGTGGACTGTGGAACACCTTGGGTTGCCTAAAGGATTCGAAGGACACTTTGTGGGCTACAGCCTCTACGTAGACGAAGTGATGGACATGTCTGGTTGCCGTGAAGAGTACATTCTGGACTCTACCGGAAAACATGTAGCGTACTTCGCGTGGTGCGTAAGCTGTGACATTCACCACAAAGGAGACATTCTGGATGTAACGTCCGTTGTCATTAATCCTGAGGCAGACTCTAAGGGCTTACAGCGATTCCTAGCGAAACGCTTTAAGTACCTTGCGGAACTCCACGATTGCGATTggg                                                                                                                                                  >  1:191326/1‑301 (MQ=255)`  
                                                                                                                                                              `ccGTTGGACTGTGGAACACCTTGGGTTGCCTAAAGGATTCGAAGGACACTTTGTGGGCTACAGCCTCTACGTAGACGAAGTGATGGACATGTCTGGTTGCCGTGAAGAGTACATTCTGGACTCTACCGGAAAACATGTAGCGTACTTCGCGTGGTGCGTAAGCTGTGACATTCACCACAAAGGAGACATTCTGGATGTAACGTCCGTTGTCATTAATCCTGAGGCAGACTCTAAGGGCTTACAGCGATTCCTAGCGAAACGCTTTAAGTACCTTGCGGAACTCCACGATTGCGATTGGgtg                                                                                                                                                >  1:295274/1‑301 (MQ=255)`  
                                                                                                                                                              `ccGGTGGACTGTGGAACACCTTGGGTTGCCTAAAGGATTCGAAGGACACTTTGTGGGCTACAGCCTCTACGTAGACGAAGTGATGGACATGTCTGGTTGCCGTGAAGAGTACATTCTGGACTCTACCGGAAAACATGTAGCGTACTTCGCGTGGTGCGTAAGCTGTGACATTCACCACAAAGGAGACATTCTGGATGTAACGTCCGTTGTCATTAATCCTGAGGCAGACTCTAAGGGCTTACAGCGATTCCTAGCGAAACGCTTTAAGTACCTTGCGGAACTCCACGATTGCGATTggggg                                                                                                                                                >  1:61799/1‑299 (MQ=255)`  
                                                                                                                                                               `cGGTGGACTGTGGAACACCTTGGGTTGCCTAAAGGATTCGAAGGACACTTTGTGGGCTACAGCCTCTACGTAGACGAAGTGATGGACATGTCTGGTTGCCGTGAAGAGTACATTCTGGACTCTACCGGAAAACATGTAGCGTACTTCGCGTGGTGCGTAAGCTGTGACATTCACCACAAAGGAGACATTCTGGATGTAACGTCCGTTGTCATTAATCCTGAGGCAGACTCTAAGGGCTTACAGCGATTCCTAGCGAAACGCTTTAAGTACCTTGCGGAACTCCACGATTGCGATTGGgtgt                                                                                                                                               <  1:59816/301‑1 (MQ=255)`  
                                                                                                                                                                      `gggctcggagatgtgtataagagacaGGAATCGAAGGACACTTTGGGGGCTACAGCCTCTACGTAGACGAAGTGATGGACATGTCTGGTTGCCGTGAAGAGTACATTCCGGACTCTACCGGAAAACATGTAGCGTACTTCGCGTGGTGCGTAAGCTGTGACATTCACCACAAAGGAGACATTATGGATGTAACGTCCGTTGTCATTAATCCTGAGGCAGACTCTAAGGGCTTACAGCGATTCCTAGCGAAACGCTTTAAGTACCTTGCGGAACTCCACGATTGCGATTGGGTGTCTCGTTg                                                                                                                                        <  1:333022/276‑1 (MQ=255)`  
                                                                                                                                                                      `cTGTGGAACACCTTGGGTTGCCTAAAGGATTCGAAGGACACTTTGTGGGCTACAGCCTCTACGTAGACGAAGTGATGGACATGTCTGGTTGCCGTGAAGAGTACATTCTGGACTCTACCGGAAAACATGTAGCGTACTTCGCGTGGTGCGTAAGCTGTGACATTCACCACAAAGGAGACATTCTGGATGTAACGTCCGTTGTCATTAATCCTGAGGCAGACTCTAAGGGCTTACAGCGATTCCTAGCGAAACGCTTTAAGTACCTTGCGGAACTCCACGATTGCGATTGGGTGTCTCGTTg                                                                                                                                        >  1:272956/1‑301 (MQ=255)`  
                                                                                                                                                                       `tgtgGAACACCTTGGGTTGCCTAAAGGATTCGAAGGACACTTTGTGGGCTACAGCCTCTACGTAGACGAAGTGATGGACATGTCTGGTTGCCGTGAAGAGTACATTCTGGACTCTACCGGAAAACATGTAGCGTACTTCGCGTGGTGCGTAAGCTGTGACATTCACCACAAAGGAGACATTCTGGATGTAACGTCCGTTGTCATTAATCCTGAGGCAGACTCTAAGGGCTTACAGCGATTCCTAGCGAAACGCTTTAAGTACCTTGCGGAACTCCACGATTGCGATTGGGTGTCTCGTTGt                                                                                                                                       <  1:199451/301‑1 (MQ=255)`  
                                                                                                                                                                        `gtgGAACACCTTGGGTTGCCTAAAGGATTCGAAGGACACTTTGTGGGCTACAGCCTCTACGTAGACGAAGTGATGGACATGTCTGGTTGCCGTGAAGAGTACATTCTGGACTCTACCGGAAAACATGTAGCGTACTTCGCGTGGTGCGTAAGCTGTGACATTCACCACAAAGGAGACATTCTGGATGTAACGTCCGTTGTCATTAATCCTGAGGCAGACTCTAAGGGCTTACAGCGATTCCTAGCGAAACGCTTTAAGTACCTTGCGGAACTCCACGATTGCGATTGTGTGTCTCGttttt                                                                                                                                      >  1:214944/1‑298 (MQ=255)`  
                                                                                                                                                                        `gtgGAACACCTTGGGTTGCCTAAAGGATTCGAAGGACACTTTGTGGGCTACAGCCTCTACGTAGACGAAGTGATGGACATGTCTGGTTGCCGTGAAGAGTACATTCTGGACTCTACCGGAAAACATGTAGCGTACTTCGCGTGGTGCGTAAGCTGTGACATTCACCACAAAGGAGACATTCTGGATGTAACGTCCGTTGTCATTAATCCTGAGGCAGACTCTAAGGGCTTACAGCGATTCCTAGCGAAACGCTTTAAGTACCTTGCGGAACTCCACGATTGCGATTGGGTGTCTCGTTGTa                                                                                                                                      >  1:159939/1‑301 (MQ=255)`  
                                                                                                                                                                            `aaCACCTTGGGTTGCCTAAAGGATTCGAAGGACACTTTGTGGGCTACAGCCTCTACGTAGACGAAGTGATGGACATGTCTGGTTGCCGTGAAGAGTACATTCTGGACTCTACCGGAAAACATGTAGCGTACTTCGCGTGGTGCGTAAGCTGTGACATTCACCACAAAGGAGACATTCTGGATGTAACGTCCGTTGTCATTAATCCTGAGGCAGACTCTAAGGGCTTACAGCGATTCCTAGCGAAACGCTTTAAGTACCTTGCGGAACTCCACGATTGCGATTGGGTGTCTCGTTGTAAGCa                                                                                                                                  <  1:39980/301‑1 (MQ=255)`  
                                                                                                                                                                             `acacCTTGGGTTGCCTAAAGGATTCGAAGGACACTTTGTGGGCTACAGCCTCTACGTAGACGAAGTGATGGACATGTCTGGTTGCCGTGAAGAGTACATTCTGGACTCTACCGGAAAACATGTAGCGTACTTCGCGTGGTGCGTAAGCTGTGACATTCACCACAAAGGAGACATTCTGGCTGTAACGTCCGTTGTCATTAATCCTGAGGCAGACTCTAAGGGCTTACAGCGATTCCTAGCGAAACGCTTTAAGTACCTTGCGGAACTCCACGATTGCGATTGGGTGTCTCGTTGTACGCAt                                                                                                                                 >  1:53821/1‑301 (MQ=255)`  
                                                                                                                                                                              `cacCTTGGGTTGCCTAAAGGATTCGAAGGACACTTTGTGGGCTACAGCCTCTACGTAGACGAAGTGATGGACATGTCTGGTTGCCGTGAAGAGTACATTCTGGACTCTACCGGAAAACATGTAGCGTACTTCGCGTGGTGCGTAAGCTGTGACATTCACCACAAAGGAGACATTCTGGATGTAACGTCCGTTGTCATTAATCCTGAGGCAGACTCTAAGGGCTTACAGCGATTCCTAGCGAAACGCTTTAAGTACCTTGCGGAACTCCACGATTGCGATTGGGTGTCTCGTTGTAAGCATg                                                                                                                                >  1:97682/1‑301 (MQ=255)`  
                                                                                                                                                                               `acCTTGGGTTGCCTAAAGGATTCGAAGGACACTTTGTGGGCTACAGCCTCTACGTAGACGAAGTGATGGACATGTCTGGTTGCCGTGAAGAGTACATTCTGGACTCTACCGGAAAACATGTAGCGCACTTCGCGTGGTGCGTAAGCTGTGACATTCACCACAAAGGAGACATTCTGGATGTAACGTCCGTTGTCATTAATCCTGAGGCAGACTCTAAGGGCTTACAGCGATTCCTAGCGAAACGCTTTAAGTACCTTGCGGAACTCCACGATTGCGATTGGttgctgtctcttatacacat                                                                                                                               >  1:321664/1‑284 (MQ=255)`  
                                                                                                                                                                                  `tgtagaagggaaagggATTGGAAGGATCCGTTGTGGGCTACAGCCTCTAATGAGACGAAGTAATGGAGATGTCTGGTTGCCGTGAAGAGTACATTCTGGACTCTACCGGAAAACATGTAGCGAAATTCGCGTGGTGCGTAAGCTGTGACATACACAACAAAGGAGACATTCTGGATGTAACGTCCGTTGTCATTAATCCTGAGGCAGACTCTAAGGGCGTACAGCGATTCGTAGCGAAACGCTTTAAGTACCTTGCGGAACTCCACGATTGCGATTGGGTGTCTCGTTGTAAGCATGAAgg                                                                                                                            <  1:183413/287‑1 (MQ=255)`  
                                                                                                                                                                                   `tGGGTTGCCTAAAGGATTCGAAGGACACTTTGTGGGCTACAGCCTCTACGTAGACGAAGTGATGGACATGTCTGGTTGCCGTGAAGAGTACATTCTGGACTCTACCGGAAAACATGTAGCGTACTTCGCGTGGTGCGTAAGCTGTGACATTCACCACAAAGGAGACATTCTGGATGTAACGTCCGTTGTCATTAATCCTGAGGCAGACTCTAAGGGCTTACAGCGATTCCTAGCGAAACGCTTTAAGTACCTTGCGGAACTCCACGATTGCGATTGGGTGTCTCGTTGTAAGCATGAAGGc                                                                                                                           <  1:207739/301‑1 (MQ=255)`  
                                                                                                                                                                                       `ttGCCTAAAGGATTCGAAGGACACTTTGTGGGCTACAGCCTCTACGTAGACGAAGTGATGGACATGTCTGGTTGCCGTGAAGAGTACATTCTGGACTCTACTGGAAAACATGTAGCGCACTTCGCGTGGTGCGTAAGCTGTGACATTCACCACAAAGGAGACATTCTGGATGTAACGTCCGTTGTCATTAATCCTGAGGCAGACTCTAAGGGCTTACAGCGATTCCT‑GCGAAACGCTTTAAGTACCTTGCGGAACTCCACGATTGCGATTGGGTGTCTCGTTGTAAGCATGAAGGCGAGAc                                                                                                                      <  1:79504/301‑1 (MQ=255)`  
                                                                                                                                                                                        `tGCCTAAAGGATTCGAAGGACACTTTGTGGGCTACAGCCTCTACGTAGACGAAGTGATGGACATGTCTGGTTGCCGTGAAGAGTACATTCTGGACTCTACCGGAAAACATGTAGCGTACTTCGCGTGGTGCGTAAGCTGTGACATTCACCACAAAGGAGACATTCTGGATGTAACGTCCGTTGTCATTAATCCTGAGGCAGACTCTAAGGGCTTACAGCGATTCCTATCGAAACGCTTTAAGTACCTTGCGGAACTCCACGATTGCGATTAGGTGTCTCGTTGTAAGCATGAAGGCGAGAc                                                                                                                      <  1:26546/301‑1 (MQ=255)`  
                                                                                                                                                                                        `tGCCTAAAGGATTCGAAGGACACTTTGTGGGCTACAGCCTCTACGTAGACGAAGTGATGGACATGTCTGGTTGCCGTGAAGAGTACATTCTGGACTCTACCGGAAAACATGTAGCGCACTTCGCGTGGTGCGTAAGCTGTGACATTCACCACAAAGGAGACATTCTGGATGTAACGTCCGTTGTCATTAATCCTGAGGCAGACTCTAAGGGCTTACAGCGATTCCTAGCGAAACGCTTTAAGTACCTTGCGGAACTCCACGATTGCGATTGGGTGTCTCGTTGTAAGCATGAAGGCGAGAc                                                                                                                      <  1:85225/301‑1 (MQ=255)`  
                                                                                                                                                                                         `gCCTAAAGGATTCGAAGGACAGTTTGTGGGCTACAGCCTCTACGTAGACGAAGTGATGGACATGTCTGGTTGCCGTGAAGAGTACATTCTGGACTCTACCGGAAAACATGTAGCGCACTTCGCGTGGTGCGTAAGCTGTGACATTCACCACAAAGGAGACATTCTGGATGTAACGTCCGTTGTCATTAATCCTGAGGCAGACTCTAAGGGCTTACAGCGATTCCTAGCGAAACGCTTTAAGTACCTTGCGGAACTCCACGATTGCGATTGGGTGTCTCGTTGTAAGCATGAAGGCGAGACa                                                                                                                     <  1:45013/301‑1 (MQ=255)`  
                                                                                                                                                                                         `gCCTAAAGGATTCGAAGGACACTTTGTGGGCTACAGCCTCTACGTAGACGAAGTGATGGACATGTCTGGTTGCCGTGAAGAGTACATTCTGGACTCTACCGGAAAACATGTAGCGTACTTCGCGTGGTGCGTAAGCTGTGACATTCACCACAAAGGAGACATTCTGGATGTAACGTCCGTTGTCATTAATCCTGAGGCAGACTCTAAGGGCTTACAGCGATTCCTAGCGAAACGCTTTAAGTACCTTGCGGAACTCCACGATTGCGATTGGGTGTCTCGTTGTAAGCATGaactgtctctt                                                                                                                     >  1:173270/1‑292 (MQ=255)`  
                                                                                                                                                                                              `aaGGATTCGAAGGACACTTTTTGGGCAACAGCCTCTACGTAGACGAAGTGATGGACATTTCTGGTTGCCGTGAAGAGTACATTCTGGACTCTACCTGAAAACATGTAGCGTACTTCGCGTGGTGCGTAAGCTGTGACATTCACCACAAAGGAGCCATTCTGGATGTAACGTCCGTTGTCATTAATCCTGAGGCAGACTCTAAGGGCTTACAGCGATTCCTAGCGAAACGCTTTAAGTACCTTGCGGAACTCCACGATTGCGATTGGGTGTCTCGTTGTAAGCATGAAGGCGAGACAATGCg                                                                                                                <  1:47983/301‑1 (MQ=255)`  
                                                                                                                                                                                              `aaGGATTCGAAGGACACTTTGTGGGCTACAGCCTCTACGTAGACGAAGTGATGGACATGTCTGGTTGCCGTGAAGAGTACATTCTGGACTCTACCGGAAAACATGTAGCGTACTTCGCGTGGTGCGTAAGCTGTGACATTCACCACAAAGGAGACATTCTGGATGTAACGTCCGTTGTCATTAATCCTGAGGCAGACTCTAAGGGCTTACAGCGATTCCTAGCGAAACGCTTTAAGTACCTTGCGGAACTCCACGATTGCGATTGGGTGTCTCGTTGTAAGCATGAAGGCGAGACAATGCg                                                                                                                <  1:73039/301‑1 (MQ=255)`  
                                                                                                                                                                                              `aaGGATTCGAAGGACACTTTGTGGGCTACAGCCTCTACGTAGACGAAGTGATGGACATGTCTGGTTGCCGTGAAGAGTACATTCTGGACTCTACCGGAAAACATGTAGCGTACTTCGCGTGGTGCGTAAGCTGTGACATTCACCACAAAGGAGACATTCTGGATGTAACGTCCGTTGTCATTAATCCTGAGGCAGACTCTAAGGGCTTACAGCGATTCCTAGCGAAACGCTTTAAGTACCTTGCGGAACTCCACGATTGCGATTGGGTGTCTCGTTGTAAGCATGAAGGCGAGACAATGCg                                                                                                                <  1:112294/301‑1 (MQ=255)`  
                                                                                                                                                                                                     `ggAAGGACACTTTGTGGGCTACAGCCTCTACGTAGACGAAGTGATGGACATGTCTTGTTGCCGTGAAGAGTACATTCTGGACTCTACCGGAAAACATGTAGCGTACTTCGCGTGGTGCGTAAGCTGTGACATTCACCACAAAGGAGACATTCTGGATGTAACGTCCGTTGTCATTAAGCCTGAGGCAGACTCTAAGGGCTTACAGCGATTCCTAGCGAAACGCTTTAAGTACCTTGCGGAACTCCACGATTGCGATTGGGTGTCTCGTTGTAAGCATGAAGGCGAGACAATGCGTGTATAc                                                                                                         <  1:242260/300‑1 (MQ=255)`  
                                                                                                                                                                                                     `ggAAGGACACTTTGTGGGCTACAGCCTCTACGTAGACGAAGTGATGGACATGTCTGGTTGCCGTGAAGAGTACATTCTGGACTCTACCGGAAAACATGTAGCGCACTTCGCGTGGTGCGTAAGCTGTGACATTCACCACAAAGGAGACATTCTGGATGTAACGTCCGTTGTCATTAATCCTGAGGCAGACTCTAAGGGCTTACAGCGATTCCTAGCGAAACGCTTTAAGTACCTTGCGGAACTCCACGATTGCGATTGGGTGTCTCGTTGTAAGCATGAAGGCGAGACAATGCGTGTATAc                                                                                                         <  1:269576/300‑1 (MQ=255)`  
                                                                                                                                                                                                     `cGAATGACACTTTGTGGGCTACAGCCTCTACGTAGACGAAGTGATGGACATGTCTGGTTTCCGTGAAGAGTACATTCTGGACTCTACCGGAAAACATGTAGCGTACTTCGCGTGGTGCGTAAGCTGTGACATTCACCACAAAGGAGACATTCTGGATGTAACGTCCGTTGTCATTAATCCTGAGGCAGACTCTAAGGGCTTACAGCGATTCCTAGCGAAACGCTTTAAGTACCTTGCGGAACTCCACGATTGCGATTGGGTGTCTCGTTGTAAGCATGAAGGCGAGACAATGCGTGTATAc                                                                                                         <  1:102764/301‑1 (MQ=255)`  
                                                                                                                                                                                                     `cGAAGGACACTTTGTGGGCTACAGCCTCTACGTAGCCGAAGTGATGGACATGTCTGGTTGCCGTGAAGAGTACCTTCTGGACTCTACCGGAAAACATGTAGCGTACTTCGCGTGGTGCGTAAGCTGTGACATTCACCACAAAGGAGACATTCTGGATGTAACGTCCGTTGTCATTAATCCTGAGGCAGACTCTAAGGGCTTACAGCGATTCCTAGCGAAACGCTTTAAGTACCTTGCGGAACTCCACGATTGCGATTGGGTGTCTCGTTGTAAGCATGAAGGCGAGACAATGCGTGTATAc                                                                                                         <  1:290170/301‑1 (MQ=255)`  
                                                                                                                                                                                                     `cGAAGGACACTTTGTGGGCTACAGCCTCTACGTAGACGAAGTGATGGACATGTCTGGTTGCCGTGAAGAGTACATTCTGGACTCTACCGGAAAACATTTAGCGTACTTCGCGTGGTGCGTAAGCTGTGACATTCACCACAAAGGAGACATTCTGGATGTAACGTCCGTTGTCATTAATCCTGAGGCAGACTCTAAGGGCTTACAGCGATTCCTAGCGAAACGCTTTAAGTACCTTGCGGAACTCCACGATTGCGATTGGGTGTCTCGTTGTAAGCATGAAGGCGAGACAATGCGTGTATAc                                                                                                         <  1:105211/301‑1 (MQ=255)`  
                                                                                                                                                                                                      `gAAGGACACTTTGTGGGCTACAGCCTCTACGTAGACGAAGTGATGGACATGTCTGGTTGCCGTGAAGAGTACATTCTGGACTCTACCGGAAAACATGTAGCGCACTTCGCGTGGTGCGTAAGCTGTGACATTCACCACAAAGGAGACATTCTGGATGTAACGTCCGTTGTCATTAATCCTGAGGCAGACTCTAAGGGCTTACAGCGATTCCTAGCGAAACGCTTTAAGTACCTTGCGGAACTCCACGATTGCGATTGGGTGTCTCGTTGTAAGCATGAAGGCGAGACAATGCGTGtatctg                                                                                                        >  1:22132/1‑298 (MQ=255)`  
                                                                                                                                                                                                            `acagttGTGGGCTACAGCCTCTACGTAGACGAAGTGATGGACATGTCTGGTTGCCGTGAAGAGTACATTCTGGACTCTACCGGAAAACATGTAGCGCACTTCGCGTGGTGCGTAAGCTGTGACATTCACCACAAAGGAGACATTCTGGATGTAACGTCCGTTGTCATTAATCCTGAGGCAGACTCTAAGGGCTTACAGCGATTCCTAGCGAAACGCTTTAAGTACCTTGCGGAACTCCACGATTGCGATTGGGTGTCTCGTTGTAAGCATGAAGGCGAGACAATGCGTGTATACTTTAagg                                                                                                  <  1:135276/297‑1 (MQ=255)`  
                                                                                                                                                                                                               `tttGTGGGCTACAGCCTCTACGTAGACGAAGTGATGGACATGTCTGGTTGCCGTGAAGAGTACATTCTGGACTCTACCGGAAAACATGTAGCGTACTTCGCGTGGTGCGTAAGCTGTGACATTCACCACAAAGGAGACATTCTGGATGTAACGTCCGTTGTCATTAATCCTGAGGCAGACTCTAAGGGCTTACAGCGATTCCTAGCGAAACGCTTTAAGTACCTTGCGGAACTCCACGATTGCGATTGGGTGTCTCGTTGTAAGCATGAAGGCGAGACAATGCGTGTATACTTTAaggagg                                                                                               <  1:150368/301‑1 (MQ=255)`  
                                                                                                                                                                                                                  `gtgGGCTACAGCCTCTACGTAGACGAAGTGATGGACATGTCTGGTTGCCGTGAAGAGTACATTCTGGACTCTACCGGAAAACATGTAGCGTACTTCGCGTGGTGCGTAAGCTGTGACATTCACCACAAAGGAGACATTCTGGATGTAACGTCCGTTGTCATTAATCCTGAGGCAGACTCTAAGGGCTTACAGTGATTCCTAGCGAAACGCTTTAAGTACCTTGCGGAACTCCACGATTGCGATTGGGTGTCTCGTTGTAAGCATGAAGGCGAGACAAGGCGTGTATTCTTTAAGGAGGtat                                                                                            >  1:139457/1‑301 (MQ=255)`  
                                                                                                                                                                                                                  `gtgGGCTACAGCCTCTACGTAGACGAAGTGATGGACATGTCTGGTTGCCGTGAAGAGTACATTCTGGACTCTACCGGAAAACATGTAGCGCACTTCGCGTGGTGCGTAAGCTGTGACATTCACCACAAAGGAGACTTTCTGGATGTAACGTCCGTTGTCATTAATCCTGAGGCAGACTCTAAGGGCTTACAGCGATTCCTAGCGAAACGCTTTAAGTACCTTGCGGAACTCCACGATTGCGATTGGGTGTCTCGTTGTAAGCATGAAGGCGAGCCAATGCGTGTATACTTTAAGGAGGtat                                                                                            >  1:257286/1‑301 (MQ=255)`  
                                                                                                                                                                                                                    `gggCTACAGCCTCTACGTAGACGAAGTGATGGACATGTCTGGTTGCCGTGAAGAGTACATTCTGGACTCTACCGGAAAACATGTAGCGTACTTCGCGTGGTGCGTAAGCTGTGACATTCACCACAAAGGAGACATTCTGGATGTAACGTCCGTTGTCATTAATCCTGAGGCAGACTCTAAGGGCTTACAGCGATTCCTAGCGAAACGCTTTAAGTACCTTGCGGAACTCCACGATTGCGATTGGGTGTCTCGTTGTAAGCATGAAGGCGAGACAATGCGTGTATACTTTAAGAAGGTATaa                                                                                          >  1:302953/1‑301 (MQ=255)`  
                                                                                                                                                                                                                     `ggCTAACGCCTCTACGTAGACGAAGTGATGGACATGTCTGGTTGCCGTGAAGAGTACATTCTGGACTCTACCGGAAAACATGTAGCGTACTTCGCGTGGTGCGTAAGCTGTGACATTCACCACAAAGGAGACATTCTGGATGTAACGTCCGTTGTCATTAATCCTGAGGCAGACTCTAAGGGCTTACAGCGATTCCTAGCGAAACGCTTTAAGTACCTTGCGGAACTCCACGATTGCGATTGGGTGTCTCGTTGTAAGCATGAAGGCGAGACAATGCGTGTATACTTTAAGGAGGTATAAg                                                                                         <  1:344333/301‑1 (MQ=255)`  
                                                                                                                                                                                                                      `gaaaagaaatgtaaggagagGAAGGGATGGACATGTCTGGTTGCCGTGAAGAGTACATGCTGGACTCTACCGGAAAACATGTAGCGTACTTCGCGTGGTGCGTAAGCTGTGACATTCACCACAAAGGAGACATTCTGGATGTAACGTCCGTTGTCATTAATCCTGAGGCAGACTCTAAGGGCTTACAGCGATTCCTAGCGAAACGCTTTAAGTACCTTGCGGAACTCAACGATTGCGATTGGGTGTCTCGTTGTAAGCATGAAGGCGAGACAATGCGTGTATACTTTAAGGAGGTATAAGt                                                                                        <  1:156722/285‑1 (MQ=255)`  
                                                                                                                                                                                                                      `gCTACAGCCTCTACGTAGACGAAGTGATTGACATGTCTGGTTGCCGTGAAGAGTACATTCTGGACTCTACCGGAAAACATGTAGCGTACTTCGCGTGGTGCGTAAGCTGTGACATTCACCACAAAGGAGACATTCTGGATGTAACGTCCGTTGTCATTAATCCTGAGGCAGACTCTAAGGGCTTACAGCGATTCCTAGCGAAACGTTTTAAGTACCTTGCGGAACTCCACGATTGCGATTGGGTGTCTCGTTGTAAGCATGAAGGCGAGACAATGCGTGTATACTTTAAGGAGGTATAAGt                                                                                        <  1:115945/301‑1 (MQ=255)`  
                                                                                                                                                                                                                      `gCTACAGCCTCTACGTAGACGAAGTGATGGACATGTCTGGTTGCCGTGAAGAGTACATTCTGGACTCTACCGGAAAACATGTAGCGCACTTCGCGTGGTGCGTAAGCTGTGACATTCACCAAAAAGGAGACATTCTGGATGTAACGTCCGTTGTCATTAATCCTGAGGCAGACTCTAAGGGCTTACAGCGATTCCTAGCGAAACGCTTTAAGTACCTTGCGGAACTCCACGATTGCGATTGGGTGTCTCGTTGTAAGCATGAAGGCGAGACAATGCGTGTATACTTTAAGGAGGTATAAGt                                                                                        <  1:250418/301‑1 (MQ=255)`  
                                                                                                                                                                                                                      `gCTACAGCCTCTACGTAGACGAAGTGATGGACATGTCTGGTTGCCATGAAGAGTACATTCTGGACTCTACCGGAAAACATGTAGCGTACTTCGCGTGGTGCGTAAGCTGTGACATTCACCACAAAGGAGACATTCTGGATGTAACGTCCGTTGTCATTAATCCTGAGGCAGACTCTAAGGGCTTACAGCGATTCCTAGCGAAACGCTTTAAGTACCTTGCGGAACTCCACGATTGCGATTGGGTGTCTCGTTGTAAGCATGAAGGCGAGACAATGCGTGTATACTTTAAGGAGGTATAAGt                                                                                        <  1:120516/301‑1 (MQ=255)`  
                                                                                                                                                                                                                      `gCTACAGCATCTACGTAGACGAAGTGATGGACATGTCTGGTTGCCGTGAAGAGTACATTCTGGACTCTACCGGAAAACATGTAGCGTACTTCGCGTGGTGCGTAAGCTGTGACATTCACCACAAAGGAGACATTCTGGATGTAACGTCCGTTGTCATTAATCCTGAGGCAGACTCTAAGGGCTTACAGCGATTCCTAGCGAAACGCTTTAAGTACCTTGCGGAACTCCACGATTGCGATTGGGTGTCTCGTTGTAAGCATGAAGGCGAGACAATGCGTGTATACTTTAAGGAGGTATAAGt                                                                                        <  1:188667/301‑1 (MQ=255)`  
                                                                                                                                                                                                                      `gCTACAGACTCTACGGAGACGAAGTGATGGACATGGCTGGTTGCCGTGAAGAGGACAGTCTGGACTCTACAGGAAAAAAGGTAGCGCACTGCGCGTGGTGAGTAAGCTGTGACATTCACCACAAAGGAGACATTCGGGATGTAACGTGAGTTGTCATTAATCCGGAGGCAGAATCTAAGGGCTTACAGCGATTCCTAGCGAAACGCTTTAAGTACCTTGCGGAACTCCACGATTGCGATTGGGTGTCTCGTTGTAAGCATGAAGGCGAGACAATGCGTGTATACTTTAAGGAGGTATAAGt                                                                                        <  1:142853/301‑1 (MQ=255)`  
                                                                                                                                                                                                                       `gacaggCCTCTACGTAGACGAAGTGATGGACATGTCTGGTTGCCGTGAAGAGTACATGCTTGACTCTACCGGAAAACATGTAGCGTACTTCGCGTGGTGCGTAAGCTGTGACATTCACCACAAAGGAGACATTCTGGATGTAACGTCCGTTGTCATTAATCCTGAGGCAGACTCTAAGGGCTTACAGCGATTCCTAGCGAAACGCTTTAAGTACCTTGCGGAACTCCACGATTGCGATTGGGTGTCTCGTTGTAAGCATGAAGGCGAGACAATGCGTGTATACTTTAAGGAGGTATAAGtt                                                                                       <  1:21182/296‑1 (MQ=255)`  
                                                                                                                                                                                                                         `aCAGCCTCTACGTAGACGAAGTGATGGACATGTCTGGTTGCCGTGAAGAGTACATTCTGGACTCTACCGGAAAACATGTAGCGTACTTCGCGTGGTGCGTAAGCTGTGACATTCACCACAAAGGAGACATTCTGGATGTAACGTCCGTTGTCATTAATCCTGAGGCAGACTCTAAGGGCTTACAGCGATTCCTAGCGAAACGCTTTAAGTACCTTGCGGAACTCCACGATTGCGATTGGGTGTCTCGTTGTAAGCATGAAGGCGAGACAATGCGTGTATACTTTAAGGCTGtatcttatac                                                                                     >  1:307842/1‑294 (MQ=255)`  
                                                                                                                                                                                                                          `cAGCCTCTACGTAGACGAAGTGATGGACATGTCTGGTTGCCGTGAAGAGTACATTCTGGACTCTACCGGAAAACATTTAGCGTACTTCGCGTGGTGCGTAAGCTGTGACATTCACCACAAAGGAGACATTCTGGATGTAACGTCCGTTGTCATTAATCCTGAGGCAGACTCTAAGGGCTTACAGCGATTCCTAGCGAAACGCTTTAAGTACCTTGCGGAACTCCACGATTGCGATTGGGTGTCTCGTTGTAAGCCTGAAGGCGAGACACTGCGTGTATACTTTAAGGAGGTATaattctgt                                                                                    >  1:165872/1‑295 (MQ=255)`  
                                                                                                                                                                                                                            `gCCTCTACGTAGACGAAGTGATGGACATGTCTGGTTGCCGTGAAGAGTACATTCTGGACTCTACCGGAAAACATGTAGCGTACTTCGCGTGGTGCGTAAGCTGTGACATTCACCACAAAGGAGACATTCTGGATGTAACGTCCGTTATCATTAATCCTGAGGCAGACTCTAAGGGCTTACAGCGATTCCTAGCGAAACGCTTTAAGTACCTTGCGGAACTCCACGATTGCGATTGGGTGTCTCGTTGTAAGCATGAAGGCGAGACAATGCGTGTATACTTTAAGGAGGTATAAGTTATggg                                                                                  >  1:338499/1‑301 (MQ=255)`  
                                                                                                                                                                                                                             `ccTCTACGTAGACGAAGTGATGGACATGTCTGGTTGCCGTGAAGAGTACATTCTGGACTCTACCGGAAAACATGTAGCGTACTTCGCGTGGTGCGTAAGCTGTGACATTCACCACAAAGGAGACATTCTGGATGTAACGTCCGTTGTCATTAATCCTGAGGCAGACTCTAAGGGCTTACAGCGATTCCTAGCGAAACGCTTTAAGTACCTTGCGGAACTCCACGATTGCGATTGGGTGTCTCGTTGTAAGCATGAAGGCGAGACAATGCGTGTATACTTTAAGGAGGTATAAGTTATGGGt                                                                                 >  1:100099/1‑301 (MQ=255)`  
                                                                                                                                                                                                                             `ccTCTACGTAGACGAAGTGATGGACATGTCTGGTTGCCGTGAAGAGTACATTCTGGACTCTACCGGAAAACATGTAGCGCACTTCGCGTGGTGCGTAAGCTGTGACATTCACCACAAAGGAGACATTCTGGATGTAACGTCCGTTGTCATTAATCCTGAGGCAGACTCTAAGGGCTTACAGCGATTCCTAGCGAAACGCTTTAAGTACCTTGCGGAACTCCACGATTGCGATTGGGTGTCTCGTTGTAAGCATGAAGGCGAGACAATGCGTGTATACTTTAAGGAGGTATAAGTTATGGGt                                                                                 >  1:22532/1‑301 (MQ=255)`  
                                                                                                                                                                                                                             `ccTCTACGTAGACGAAGTGATGGACATGTCTGGTTGCCGTGAAGAGTACATTCTGGACTCTACCAGAAAACATGTAGCGTACTTCGCGTGGTGCGTAAGCTGTGACATTCACCACAAAGGAGACATTCTGGATGTAACGTCCGTTGTCATTAATCCTGAGGCAGACTCTAAGGGCTTACAGCGATTCCTAGCGAAACGCTTTAAGTACCTTGCGGAACTCCACGATTGCGATTGGGTGTCTCGTTGTAAGCATGAAGGCGAGACAATGCGTGTATACTTTAAGGAGGTATAAGTTATGGGt                                                                                 >  1:163958/1‑301 (MQ=255)`  
                                                                                                                                                                                                                                `ctACGTAGACGAAGTGATGGACATGTCTGGTTGCCGTGAAGAGTACATTCTGGACTCTACCGGAAAACATGTAGCGTACTTCGCGTGGTGCGTCAGCTGTGACCTTCACCCCAAAGGAGACATTCTGGATGTAACGTCCGTTGTCATTAATCCTGAGGCAGACTCTAAGGGCTTACAGCGATTCCTAGCGAAACGCTTTCAGTACCTTGCTGAACTCCACGATTGCGATTGGGTGCCTCGTTGTAAGCATGACGACGAGACAATGCGTTTCTACTTTAAGTAGGACTACGTTATGTTTAAg                                                                              >  1:145351/1‑301 (MQ=255)`  
                                                                                                                                                                                                                                                                        `gagTACATTCTGGACTCTACCGGAAAACATGTAGCGTACTTCGCGTGGTGCGTAAGCTGTGACATTCACCACAAAGGAGACATTCTGGATGTAACGTCCGTTGTCATTAATCCTGAGGCAGACTCTAAGGGCTTACAGCGATTCCTAGCGAAACGCTTTAAGTACCTTGCGGAACTCCACGATTGCGATTGGGTGTCTCGTTGTAAGCATGAAGGCGAGACAATGCGTGTATACTTTAAGGAGGTATAAGTTATGGGTAAGAAAGTTAAGAAGGCCGTgaagctgtctcttatacacatct                                      >  1:22755/1‑282 (MQ=255)`  
                                                                                                                                                                                                                                                                          `gTACATTCTGGACTCTACCGGAAAACATGTAGCGTACTTCGCGTGGGGCGTAAGCTGTGACATTCACCACAAAGGAGACATTCTGGATGTAACGTCCGTTGTCATTAATCCTGAGGCAGACTCTAAGGGCTTACAGCGATTCCTAGCGAAACGCTTTAAGTACCTTGCGGAACTCCACGATTGCGATTGGGTGTCTCGTTGTAAGCATGAAGGCGAGACAATGCGTGTATACTTTAAGGAGGTATAAGTTATGGATAAGAAAGTTAAGAAGGCCGTGAAGAAAGTCACCAAGTCCGTTAAg                                    <  1:278937/301‑1 (MQ=255)`  
                                                                                                                                                                                                                                                                            `aCATTCTGGACTCTACCTGAAAACATGTAGCGCAATTCGCGTGGTGCGTAAGCTGTGACATTCACCACAAAGGAGACATTCTGGATGTAACGTCCGTTGTCATTAATCCTGAGGCAGACTCTAAGGGCTTACAGCGATTCCTAGCGAAACGCTTTAAGTACCTTGCGGAACTCCACGATTGCGATTGGGTGTCTCGTTGTAAGCATGAAGGCGAGACAATGCGTGTATACTTTAAGGAGGTATAAGTTATGGGTAAGAAAGTTAAGAAGGCCGTGAAGAAAGTCACCAAGTCCGTTAAGaa                                  <  1:127605/301‑1 (MQ=255)`  
                                                                                                                                                                                                                                                                                  `tGGACACTACCGGAAAACATGTAGCGCACTTCGCGTGGTGCGTAAGCTGTGACATTCACCACAAAGGAGACATTCTGGATGTAACGTCCGTTGTCATTAATCCTGAGGCAGACTCTAAGGGCTTACAGCGATTCCTAGCGAAACGCTTTAAGTACCTTGCGGAACTCCACGATTGCGATTGGGTGTCTCGTTGTAAGCATGAAGGCGAGACAATGCGTGTATACCTTAAGGAGGTATAAGTTATGGGTAAGAAAGTTAAGAAGGCCGTGAAGAAAGTCACCAAGTCCGTTAAGAAAGTCGt                            <  1:310746/301‑1 (MQ=255)`  
                                                                                                                                                                                                                                                                                          `aCCGGAAAACATGTAGCGTACTTCGCGTGGTGCGTAAGCTGTGACATTCACCACAAAGGAGACATTCTGGATGTAACGTCCGTTGTCATTAATCCTGAGGCAGACTCTAAGGGCTTACAGCGATTCCTAGCGAAACGCTTTAAGTACCTTGCGGAACTCCACGATTGCGATTGGGTGTCTCGTTGTAAGCATGAAGGCGAGACAATGCGTGTATACTTTAAGGAGGTATAAGTTATGGGTAAGAAAGTTAAGAAGGCGGTGAAGAAAGTCACCAAGTCCGTTAAGAAAGTCGTTaaggaag                    >  1:197250/1‑301 (MQ=255)`  
                                                                                                                                                                                                                                                                                          `aCCGGAAAACATGTAGCGTACTTCGCGTGGTGCGTAAGCTGTGACATTCACCACAAAGGAGACATTCTGGATGTAACGTCCGTTGTCATTAATCCTGAGGCAGACTCTAAGGGCTTACAGCGATTCCTAGCGAAACGCTTTAAGTACCTTGCGGAACTCCACGATTGCGATTGGGTGTCTCGTTGTAAGCATGAAGGCGAGACAATGCGTGTATACTTTAAGGAGGTATAAGTTATGGGTAAGAAAGTTAAGAAGGCCGTGAAGAAAGTCACCAAGTCCGTTAAGAAAGTCGTTaaggaag                    >  1:246879/1‑301 (MQ=255)`  
                                                                                                                                                                                                                                                                                                  `aCATGTAGCGCACTTCGCGTGGTGCGTAAGCTGTGACATTCACCACAAAGGAGACATTCTGGATGTAACGTCCGTTGTCATTAATCCTGAGGCAGACTCTAAGGGCTTACAGCGATTCCTAGCGAAACGCTTTAAGCACCTTGCGGAACTCCACGATTGCGATTGGGTGTCTCGTTGTAAGCATGAAGGCGAGACAATGCGTGTATACTTTAAGGAGGTATAAGTTATGGGTAAGAAAGTTAAGAAGGCCGTGAAGAAAGTCACCAAGTCCGTTAAGAAAGTCGTTAAGGAAGGGGCTCGt            >  1:8273/1‑301 (MQ=255)`  
                                                                                                                                                                                                                                                                                                        `aGCGTACTTCGCGTGGTGCGTAAGCTGTGACATTCACCACAAAGGAGACATTCTGGATGTAATGTCCGTTGTCATTAATCCTGAGGCAGACTCTAAGGGCTTACAGCGATTCCTAGCGAAACGCTTTAAGTACCTTGCGGAACTCCACGATTGCGATTGGGTGTCTCGTTGTAAGCATGAAGGCGAGACAATGAGTGTATACTTTAAGGAGGTATAAGTTATGGGTAAGAAAGTTAAGAAGGCCGTGAAGAAAGTCACCAAGTCCGTTAAGAAAGTCGTTAAGGAAGGGGCTCGTCCGGtt      >  1:153796/1‑301 (MQ=255)`  
                                                                                                                                                                                                                                                                                                        `aGCGTACTTCGCGTGGTGCGTAAGCTGTGACATTCACCACAAAGGAGACATTCTGGATGTAACGTCCGTTGTCATTAATCCTGAGGCAGACTCTAAGGGCTTACAGCGATTCCTAGCGAAACGCTTTAAGTACCTTGCGGAACTCCACGATTGCGATTGGGTGTCTCGTTGTAAGCATGAAGGCGAGACAATGCGTGTATACTTTAAGGAGGTATAAGTTATGGGTAAGAAAGTTAAGAAGGCCGTGAAGAAAGTCACCAAGTCCGTTAAGAAAGTCGTTAAGGAAGGGGCTCGTCCGGtt      >  1:201800/1‑301 (MQ=255)`  
                                                                                                                                                                                                                                                                                                         `gCGTACTTCGCGTGGTGCGTAAGCTGTGACATTCACCACAAAGGAGACATTCTGGATGTAACGTCCGTTGTCATTAATCCTGAGGCAGACTCTAAGGGCTTACAGCGATTCCTAGCGAAACGCTTTAAGTACCTTGCGGAACTCCACGATTGCGATTGGGTGTCTCGTTGTAAGCATGAAGGCGAGACAATGCGTGTATACTTTAAGGAGGTATAAGTTATGGGTAAGAAAGTTAAGAAGGCCGTGAAGAAAGTCACCAAGTCCGTTAAGAAAGTCGTTAAGGAAGGGGCTCGTCCGGTTa     >  1:43024/1‑301 (MQ=255)`  

                                                                                                                                                                                                                                                                                                            `|`                                                                                                                                                                                                                                                                                                              
`AGGCCTCAAGGTCGAACGTGATGATTCCGTAGCGAAGTCTAAAGCCAGCGGCAATGGCTCCGCTCGTATGAAATCTTCCATCCGTAAGTCCGCATTTGGAGGTAAGAAGTGATGTCTGAGTTCACATGTGTGGAGGCTAAGAGTCGCTTCCGTGCAATCCGGTGGACTGTGGAACACCTTGGGTTGCCTAAAGGATTCGAAGGACACTTTGTGGGCTACAGCCTCTACGTAGACGAAGTGATGGACATGTCTGGTTGCCGTGAAGAGTACATTCTGGACTCTACCGGAAAACATGTAGCGTACTTCGCGTGGTGCGTAAGCTGTGACATTCACCACAAAGGAGACATTCTGGATGTAACGTCCGTTGTCATTAATCCTGAGGCAGACTCTAAGGGCTTACAGCGATTCCTAGCGAAACGCTTTAAGTACCTTGCGGAACTCCACGATTGCGATTGGGTGTCTCGTTGTAAGCATGAAGGCGAGACAATGCGTGTATACTTTAAGGAGGTATAAGTTATGGGTAAGAAAGTTAAGAAGGCCGTGAAGAAAGTCACCAAGTCCGTTAAGAAAGTCGTTAAGGAAGGGGCTCGTCCGGTTAAAC  >  NC_001604.1/19019‑19619`

  

Alignment Legend

Aligned base mismatch/match (shaded by quality score): `ATCG/ATCG` `< 3 ≤` `ATCG/ATCG` `< 11 ≤` `ATCG/ATCG` `< 24 ≤` `ATCG/ATCG` `< 36 ≤` `ATCG/ATCG` `< 38 ≤` `ATCG/ATCG`

Unaligned base: `atcg`    Masked matching base: `atcg`    Alignment gap: `‑`    Deleted base: `‑`
